# One-Dimensional ZnO/Gold Junction for Simultaneous and Versatile Multisensing Measurements

**DOI:** 10.1038/srep29763

**Published:** 2016-07-13

**Authors:** Beatrice Miccoli, Valentina Cauda, Alberto Bonanno, Alessandro Sanginario, Katarzyna Bejtka, Federico Bella, Marco Fontana, Danilo Demarchi

**Affiliations:** 1 Department of Electronics and Telecommunication, Politecnico di Torino, Corso Duca degli Abruzzi 24, Torino 10129, Italy; 2 Center for Space Human Robotics@PoliTo, Istituto Italiano di Tecnologia, Corso Trento 21, Torino 10129, Italy; 3 Department of Applied Science and Technology, Politecnico di Torino, Corso Duca degli Abruzzi 24, Torino 10129, Italy

## Abstract

The sensing capabilities of zinc oxide nano/micro-structures have been widely investigated and these structures are frequently used in the fabrication of cutting-edge sensors. However, to date, little attention has been paid to the multi-sensing abilities of this material. In this work, we present an efficient multisensor based on a single zinc oxide microwire/gold junction. The device is able to detect in real time three different stimuli, UV-VIS light, temperature and pH variations. This is thanks to three properties of zinc oxide its photoconductive response, pyroelectricity and surface functionalization with amino-propyl groups, respectively. The three stimuli can be detected either simultaneously or in a sequence/random order. A specific mathematical tool was also developed, together with a design of experiments (DoE), to predict the performances of the sensor. Our micro-device allows reliable and versatile real-time measurements of UV-VIS light, temperature and pH variations. Therefore, it shows great potential for use in the field of sensing for living cell cultures.

The integration of nanostructured materials on microelectronic chips is essential to achieve highly controlled bottom-up processes leading to high-performing devices, i.e. sensors^1^,^2^,^3^, energy harvesters^4^,^5^,^6^, non-volatile memories^7^,^8^, and field-effect transistors^2^,^8^. Recent studies have reported on the integration of various micro- and nanostructures on micropatterned electrodes. Relevant examples are p-type field effect transistors using CuO nanowires (NWs)^2^, photoconductive-terahertz detectors with single GaAs NWs^3^, electromechanical devices with ZnO microwires (μ-wires)^9^, photodetectors based on SiC and ZnO NWs^10^,^11^.

In particular ZnO nano/micro-structures have attracted a lot of interest due to their potential application in a wide range of devices, such as laser and light-emitting diodes^12^, piezoelectric generators and transducers^4^,^5^, gas^13^,^14^ and pH sensors^15^,^16^, as well as ultraviolet (UV) photodetectors^17^,^18^ and protein biosensors^19^.

Most of the literature studies exploit the sensing effect of a multitude of ZnO wires. ZnO wires can be vertically aligned or randomly dispersed between the patterned metal electrodes or sandwiched between them. In general, devices using a multitude of nano/micro-wires offer high signals due to the large sensing area available. Usually, they require a fine tuning of the synthetic approach and deposition processes in multiple steps that may include growth, sonication, and distribution of the nano/micro-wires on the pre-patterned electrodes^20^,^21^. Alternatively, the integration of single sensing structures on a microelectronic chip^10^,^22^ offers an extremely sensitive detection capability, even able to detect single molecules, thereby improving the limit-of-detection. However, the positioning of the sensing structures and their alignment on electrodes is a challenging process if random dropping or pick-and-place methods are used. For example, time-consuming and expensive equipment is necessary to place randomly dispersed structures on electrodes^23^, also preventing any control over the reproducibility of the final device and the related measurements. On the contrary, the dielectrophoresis (DEP) process offers a more reproducible, faster, easier-to-handle and cheaper method to align single wires between two facing electrodes^15^,^16^,^22^,^24^,^25^,^26^. DEP was used in the present study to align single functionalized ZnO μ-wires (200–600 nm in diameter and 5–15 μm in length) directly onto a pre-fabricated gold electrode-array chip.

Currently, most of the literature focuses on arrays of ZnO nanostructures and it reports mainly on their light and gas sensing properties^14^,^20^. Although few papers present the electrical dependence of ZnO on pH and temperature^1^,^27^, Menzel *et al*.^28^ showed the measurement of UV, pH and temperature stimuli using arrays of vertically aligned NWs. These measurements were not carried out simultaneously in one single experiment. By contrast, our innovative work focused on exploiting the multisensing behavior of a single ZnO μ-wire under ultraviolet-visible (UV-VIS) light, and at specific temperatures and pH values, within the same experiment. In particular, the stimuli-responsive measurements of these three parameters were taken both simultaneously and in a random sequence on the same chip. Embedding this multisensor in living cell culture microenvironments could allow the precise monitoring of the three aforementioned parameters, which are crucial for cell life. Variations in pH are strongly related to enzyme activity and localization, as well as taking part in the process of ATP synthesis^29^. Temperature directly affects the molecular architecture and mechanical properties of cells. Moreover, the cellular membrane permeability strongly depends on temperature, making this parameter of crucial importance for the study of intra/extra-cellular therapeutic treatments^30^. Finally, the tuning and monitoring of UV radiation directly impact on cell survival and they are of extreme importance in different processes of microorganism disinfection^31^.

The aim of this work was to develop a multisensor whose transduction mechanism is represented by a single ZnO μ-wire/gold junction. This multisensor will be used to detect in real-time the three different stimuli, UV-VIS light, temperature and pH variations, vital for cell culture growth and survival. In fact, the results obtained by this first proof-of-concept device will underpin further work on this topic, leading to a final multisensor integrating living cells in which nanotechnology and electronics meet biological living systems.

## Results

### Multiparametric sensor assembling

**Figure 1:**
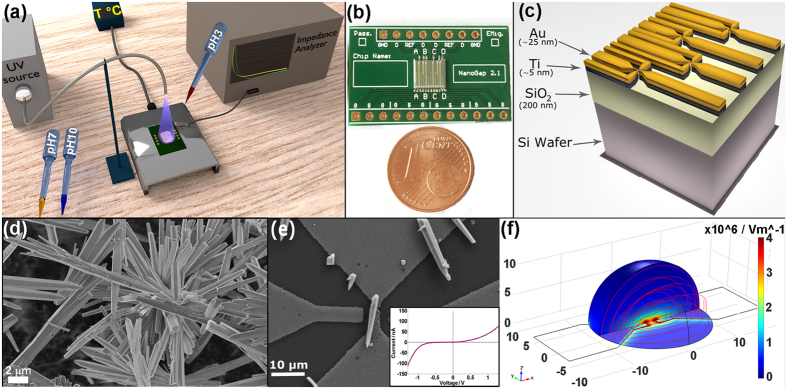
Setup of the ZnO μ-wire/gold based multifunctional sensor. (**a**) Schematic view of the multisensing experimental setup. (**b**) The final chip assembled onto custom PCB and (**c**) its cross-sectional scheme showing the patterned gold electrodes. FESEM image of (**d**) the bare ZnO μ-wires in powder form and of (**e**) a single amine-functionalized ZnO μ-wire bridging the gold microelectrodes, obtaining a non-linear I-V characteristic (inset). (**f**) Simulation (COMSOL Multiphysics) of the electric field between the gold electrodes during DEP.

The measurement setup of our multiparametric sensing platform is outlined in Fig. 1a. The setup allows simultaneous stimulation of a chip with single ZnO μ-wire/gold junctions under varying UV-VIS light, temperature and pH conditions. The array of gold electrodes, where the ZnO μ-wires were positioned and investigated, was prepared by conventional photolithography. Four gold butterfly probes (Fig. 1b) were deposited on a Ti/SiO_2_/Si wafer (5 × 5 mm) (Fig. 1c) as reported in the Methods section and in Cauda *et al*.^15^. The chip was wire bonded to a printed circuit board (PCB) and a thin layer of epoxy resin protected the bonds. The gold probes were broken to obtain four couples of facing electrodes of 0.5–1 μm width on the same chip by using the electromigration-induced break junction (EIBJ) method. The EIBJ is controlled by a customized embedded modular electronic system^32^. Each electrode pair could be individually accessed for DEP and electrical characterization. The system configuration thus enabled independent ZnO μ-wire positioning and measurements to be performed on each of the four electrode pairs on the same chip. The ZnO μ-wires were synthesized by a simple low-temperature hydrothermal route (Fig. 1d) and they crystallized in the typical wurtzite structure, as confirmed by the X-ray diffraction shown in Fig. S1 of the Supplementary Information (S.I.). The ZnO μ-wires were then chemically functionalized with aminopropyltrimethoxysilane to improve the pH sensitivity and prevent the dissolution of the μ-wires when in contact with strong basic and acid pH^15^,^32^. Evidence of the successful chemical functionalization was proven by infrared spectroscopy (see Fig. S2 in the S.I.). A diluted solution of μ-wires in isopropanol was dropped on the chip at the same time as the application of the DEP signal. The DEP process enabled the efficient alignment (84%) of functionalized ZnO μ-wires on the four electrodes of 31 chips, therefore on 124 electrode pairs (see Supplementary Fig. S3). An alternating electric field was applied to the electrodes to attract the ZnO μ-wire to the region with the highest electric field (positive DEP effect Fig. 1f). A representative result of a DEP process, observed with a field emission scanning electron microscope (FESEM), is outlined in Fig. 1e and others are reported in Supplementary Fig. S4. This method allows high chip re-usability, since the μ-wires can be quickly removed by sonication.

Once the individual ZnO μ-wires were aligned on each gold-electrode pair, forming a ZnO/gold junction, the electrical characterization in DC returned in all cases a non-linear I-V characteristic (inset of Fig. 1e), i.e. a Schottky behavior with currents of hundreds of nA or few μA, as previously reported^15^,^16^. Further details about the ZnO/gold Schottky junction are outlined in Supplementary Note 1. AC measurements were performed with a precision impedance analyzer (4294a by Keysight Technologies) from 5 to 100 kHz and assuming that each ZnO/gold junction had a capacitor and a resistor in parallel^27^.

**Figure 2:**
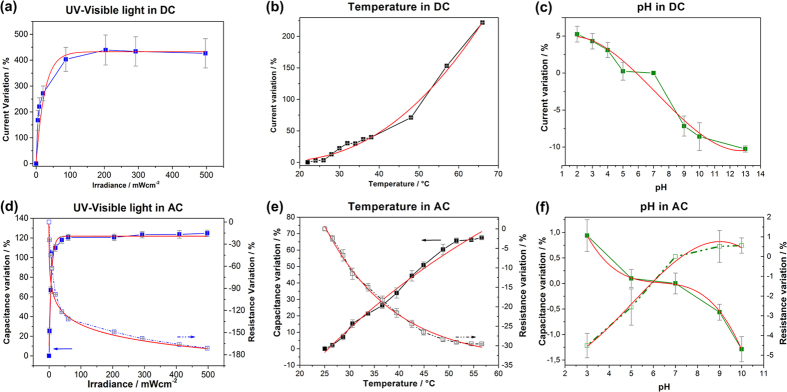
DC and AC electrical characterization of individual stimuli. The electrical behavior of the ZnO-gold junction measured under (**a**,**d**) UV-VIS irradiation, (**b**,**e**) temperature and (**c**,**f**) pH variations. Top graphs are obtained in DC domain, measuring the current % variation with respect to the starting conditions at 1 V. Bottom graphs are AC measurements with capacitance (left y-axis) and resistance (right y-axis) % variations obtained at a fixed frequency of 5 kHz. Standard deviation bars are also reported for each data set. The experimental data (lines with symbol) are fitted by the red curves.

To properly interpret the sensing measurement results, the influence of three different parameters (UV-VIS light, temperature and pH) were initially evaluated separately on single ZnO/gold junctions, both with DC and AC analyses (Fig. 2). Control experiments, for each external stimulus, when no ZnO μ-wire was bridging the gold electrodes, are reported in Supplementary Fig. S5. For an effective comparison of the results and further modeling, the electrical values in Fig. 2 are reported as a percentage of the initial value (i.e., dark condition, 25 °C and pH 7). The absolute values of current, capacitance and resistance are shown in Supplementary Figs S6–S9.

### UV-VIS light characterization

The UV-VIS light incident perpendicularly on a single ZnO/gold junction abruptly increased the measured current from dark to light conditions. By varying the UV-VIS irradiation from 0 up to 450 mW·cm^−2^, at a fixed voltage of 1 V, the DC current increased to 450% of the reference value measured in the dark (Fig. 2a). A similar behavior was observed for the AC measurements under UV-VIS irradiation, in the frequency range 5–100 KHz. Specifically, the capacitance increased and the resistance decreased as outlined in Fig. 2d, which shows measurements at 5 KHz (the measurements at the remaining frequencies are reported in Supplementary Fig. S7). Small values of UV-VIS irradiance can be discerned (down to 0.1 mW∙cm^−2^) thanks to the selected frequency range within which the measurements are not affected by Flicker noise (1/f noise). Flicker noise afflicts electronic devices and materials at low frequencies, but it can be significantly reduced by performing measurements at high frequencies. In contrast, DC measurements are at low frequency and hence suffer from higher noise and are only reliable for higher irradiance values. UV-VIS light on/off cycles were carried out to further assess the photo-response of a single ZnO μ-wire (see Supplementary Fig. S10). The response was extremely fast and it was possible to clearly distinguish between the “on” and “off” states.

The photosensitivity of ZnO nanostructures is attributed to electron-hole pairs generation due to the incident photons^17^,^20^,^23^. At increasing irradiance values, more electron-hole pairs are generated. Holes are trapped by the negatively-charged oxygen ions (O^2−^) adsorbed on the ZnO surface, whereas the electrons are available in the conduction band, contributing to a current increase. The current plateau observed in Fig. 2a,d, at higher light irradiance, may be attributed to the limited number of O^2−^ adsorption sites on the ZnO surface, being saturated by the trapped holes. This leads to a higher recombination rate of electron-hole pairs and thus to a reduction of carriers available for conduction. This behavior highlights the greater sensitivity of ZnO at low irradiance values than at higher ones. Between 0 and 50 mW∙cm^−2^ a sensitivity of 0.03 μA∙mW^−1^∙cm^2^ was estimated for the ZnO/gold junction, whereas it fell to 1.70∙10^−4 ^μA∙mW^−1^∙cm^2^ approximately at 150 mW∙cm^−2^ (further details about the sensitivity computation are reported in Supplementary Fig. S6a). The presence of amine-functionalizing groups on the surface is not believed to interact with the electron-hole pair formation and, at worst, it can slightly decrease the photoresponse with respect to a pristine ZnO material. In addition, the current increase due to local heating provided by the UV-VIS source (about 0.5–1 °C) can be neglected during irradiation experiments. In fact, no further current increase was observed by maintaining a constant irradiation over time on the ZnO/gold junction (see Supplementary Fig. S11), meaning that the dominant effect reported in Fig. 2a,d is the photoelectric one.

### Temperature sensing

Concerning the temperature variation, an exponential increase in both the current in DC and the capacitance in AC (with a concomitant decrease in resistance) was observed (Fig. 2b,e). These variations, at increasing temperature, are attributed to the coupling of the semiconducting and pyroelectric properties of ZnO material. Specifically, a polarization electric field is created along the μ-wire length as a result of the temperature variation over time^27^,^33^. Due to the Schottky barriers between the ZnO and Au-electrodes, and to the temperature increase, an electric potential is established between the two ends of the ZnO μ-wire, resulting in a positively and a negatively charged end. The negative electric potential (V^−^) can raise the local conduction band of ZnO and the Fermi level of one gold electrode by ΔE = e(V^+^−V^−^). This drives electrons to flow from one gold electrode to the other through the external electric circuit (i.e., the PCB board and the connecting wires) thus leading to the current measurement. As it is a pyroelectric material, currents are only generated by ZnO during the temperature variation, whereas when the equilibrium is reached at the new temperature no current output is observed^33^.

The conduction mechanism of ZnO/gold Schottky junctions is currently under study. However, as a first approximation, the experimental I-V curves are compared with the behavior of an ideal Schottky diode at different temperatures, considering the thermionic emission model (see Supplementary Fig. S12). Specifically, the obtained ideality factor is 10.54–9.65 in the temperature range 30–105 °C. Furthermore, the temperature dependence of the barrier height (1.5–1.8 V at 30–105 °C) highlights a strong influence of additional mechanisms that are not considered in the thermionic model, such as the field emission and the thermionic-field emission ones (see Supplementary Fig. S13 and Supplementary Note 1). Both these effects could be connected to quantum mechanical tunneling through the potential barrier between the metal and the semiconductor^34^,^35^. The observed deviation from the ideal Schottky diode model may be attributed to several factors, i.e. surface-localized states due to the high number of defects present on the μ-wire surface, barrier height inhomogeneities, Fermi level pinning, and tunneling effects^35^.

### pH detection

pH-dependent DC measurements, on a single ZnO/gold junction, showed a current decrease when the pH was increased from 2 to 13 (Fig. 2c). The conductance of the ZnO μ-wire varied almost linearly when the pH was varied from 2 to 5 and the sensitivity extracted from the curve slope is −4.5 nA·pH^−1^ (or −4.8 nS∙pH^−1^ as depicted in Supplementary Fig. S6c and according to the literature)^36^. Similarly, during the AC measurements the capacitance decreased and the resistance increased as the pH was increased from 3 to 10 (Fig. 2f). The prominent role in pH sensing is here played by the aminopropyl-group (NH_2_) functionalization on the ZnO surface. This is because the aminopropyl-group is protonated from -NH_2_ to -NH_3_^+^ in acidic media and it is, instead, deprotonated when in contact with basic media^15^,^16^. Besides, also the ZnO surface undergoes protonation and deprotonation mechanisms, which e.g., leads to the formation of hydroxyl groups at acidic pH values. However, the large amount of amine-functionalizing groups was responsible for the majority of the conductance variation of the ZnO/gold junction as also quantitatively demonstrated by Cauda *et al*.^15^. In details, the pH-dependent net surface charge changes the voltage at the ZnO/liquid interface and this modifies both the DC current and the impedance in the AC mode^15^,^16^,^36^. It is noteworthy that no evidence of degradation was observed on the ZnO μ-wires after pH-related experiments. This was due to the protective role of the aminopropyl-groups, grafting in large amount the surface of ZnO (around 1.78 molecules∙nm^−2^)^15^.

### Multiparametric sensor characterization

The results obtained when the UV-VIS light, temperature and pH were varied one by one were observed on distinct amine-functionalized ZnO/gold junctions (see Table 1), thus strengthening the repeatability of the proposed sensing mechanism. In addition, a mathematical modeling of the experimental trends was also performed. This mathematical modeling was able to properly fit the measured data (Supplementary Table 1) with reproducible exponential or polynomial curves (red lines in Fig. 2).

**Figure 3:**
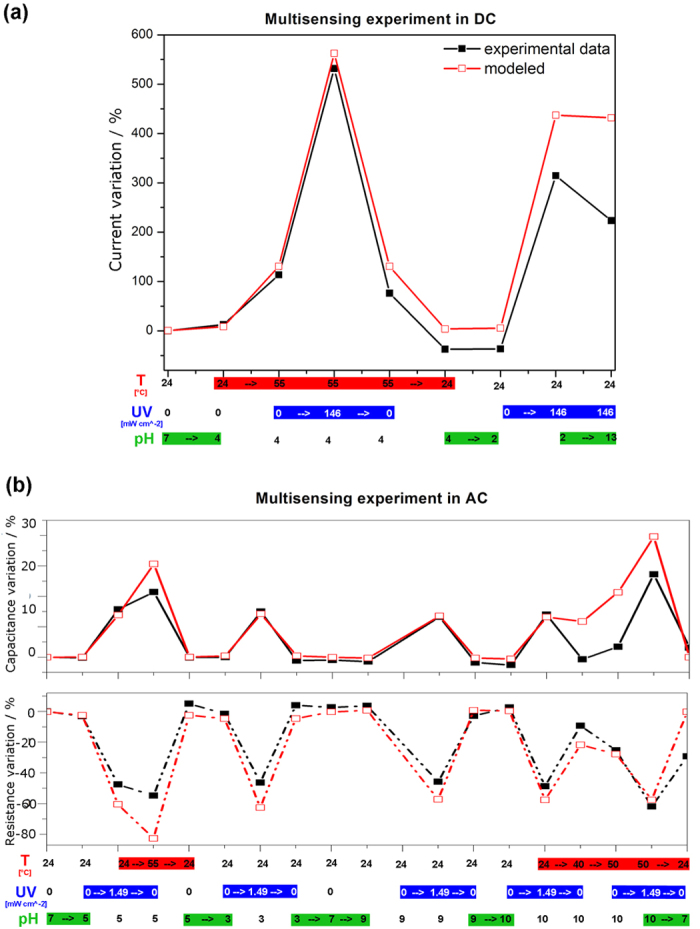
DC and AC characterization of the multifunctional sensor. Multisensing measurements on ZnO/gold junctions by varying UV-VIS irradiance, temperature and pH simultaneously and in a random order within different ranges in both (**a**) DC domain at 1 V and (**b**) AC one at 5 kHz.

These results allow to use the ZnO μ-wire/gold junction as a multisensing device. In light of this, the UV-VIS light, temperature and pH stimuli were applied in sequence or simultaneously on various ZnO/gold junctions. DC and AC real-time outputs (Fig. 3), measured on two different junctions respectively, were acquired starting from room temperature, neutral pH and dark conditions. Successively, the three stimuli were varied independently, resulting in a sum of effects when two or three parameters were changed simultaneously. The colored bars underneath the graphs in Fig. 3 show in which order the three stimuli were applied and modified. Additional details about the signal-to-noise ratio (SNR) of both one by one sensing measurements and multisensing ones can be found in Supplementary Note 2 and Supplementary Figs S16–S18.

The DC and AC outputs of the ZnO/gold junction acquired during the multisensing experiments were then analytically reconstructed. The analytical reconstruction was achieved by a linear combination of the fitting equations which regulate the electrical output when the external stimuli are varied one by one (see equations (1)–(3)). Thus, the output trends of the multisensor, both in DC and AC, was analytically created as depicted Fig. 3 (red lines).


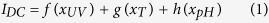



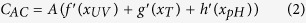



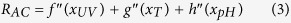


In equations (1–3), *f, f* ′, *f* ″, *g, g*′, *g″, h, h*′, *h″* are the modeling equations when the three external stimuli are varied one by one. Therefore they predict the electrical output as a function of UV-VIS irradiance (x_UV_), temperature (x_T_) and pH (x_pH_) (see also Supplementary Note 3). The coefficient *A* is a corrective factor (calculated to be 0.28) ascribing the influence of other capacitance variations, external to the ZnO/gold junction, on the experimental behavior upon the stimuli application. These external contributions are instead negligible on the resistance (and thus on the current) value of the multisensor.

Since the modeling of multisensors is a challenging task, considering a linear combination of equations (1)–(3) as all-inclusive model is a simplification. Nonetheless, the multisensing fitting curves, *I*_*DC*_, *R*_*AC*_ and *C*_*AC*_, reasonably predict the experimental behaviors considering that the modeling equations were obtained from different junctions and with a simplified global model. Only slight deviations appear when the three stimuli are combined together due to the propagation of the fitting errors (see Supplementary Note 3). Therefore, both the predicted and the experimental curves can be generalized for all our chips, despite different μ-wire shapes, dimensions or contact area with the underlying gold electrodes. This is not a trivial effort considering that the synthesized ZnO μ-wires cannot have all the same size, hence leading to slightly different values of resistance and capacitance. Moreover, the obtained multiparametric equations (1)–(3) can reconstruct back the sensing behavior in both AC and DC domains under a specific external stimulus by fixing two boundary conditions (see Supplementary Figs S14 and S15). We can thus predict the behavior of the sensor output, both in AC and in DC, without performing any experiment.

### DoE

In the previous section, we proposed a linear combination of fitting equations to model the behavior of the multisensor under different stimuli. Now we would like to offer a deeper statistical analysis of our system based on a multivariate chemometric approach. In particular, a design of experiments (DoE) is here proposed to correlate the measured experimental parameters to their multiparametric set-up that simultaneously varies temperature, light intensity and pH. These latter form an experimental domain that can be investigated by means of a D-Optimal design, which represents a regression model described by the equation:


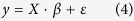


where *y* is a (*N* × 1) vector of observed responses (i.e., capacitance, resistance, current), *X* is a (*N* × *p*) extended design matrix, containing the *n* experimental runs extended with additional columns for *p* terms of the model (i.e., constant term, interaction terms, square terms, …), *β* is a (*p* × 1) vector of unknown coefficients to be determined by fitting the model to the observed responses, *ε* is a (*N* × 1) vector of residuals (the differences between the observed and predicted values of the response *y*). In summary, it is possible to exactly identify which is the singular contribution of each parameter (temperature, light intensity and pH) on the measured responses (capacitance, resistance, current).

**Figure 4:**
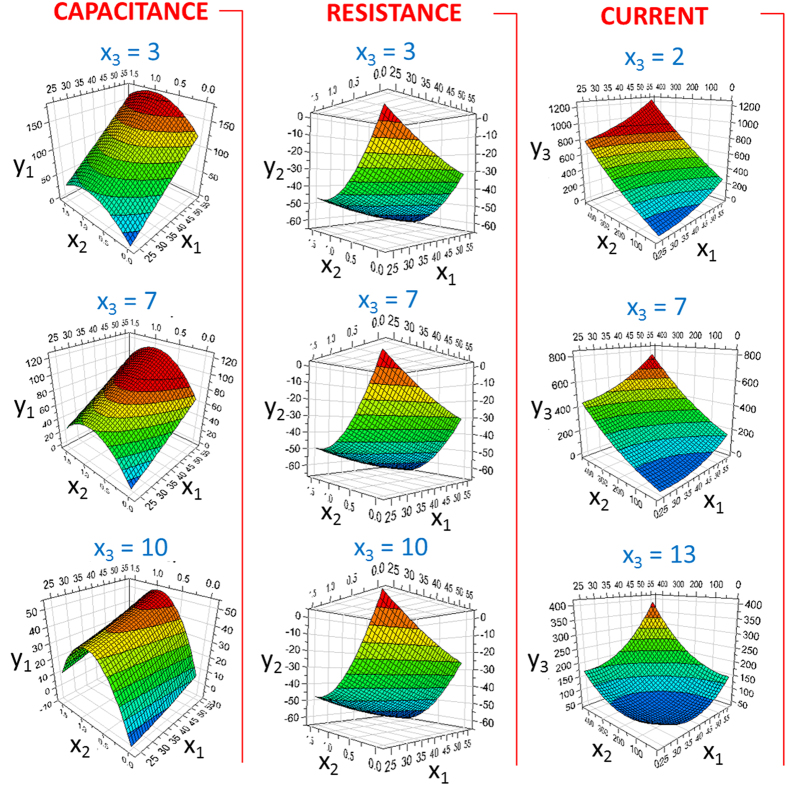
Fitted response surfaces for the multivariate chemometric model. The isoresponse plots show the effect of the experimental variables (*x*_1_ = T in °C, *x*_2_ = UV in mW∙cm^−2^, *x*_3_ = pH) of the measured responses (*y*_1_ = capacitance, *y*_2_ = resistance, *y*_3_ = current).

Assisted by the software MODDE (see the Experimental section for details), 18 experiments were identified in order to explore the whole experimental domain of each of the three variables. These experiments were performed and the D-Optimal model was fitted through a multiple linear regression. As a result of this fitting process, an empirical relationship between the measured response and the variables can be expressed in polynomial form:


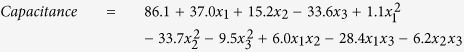



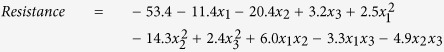



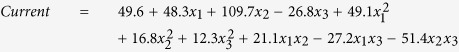


where *x*_1_ = T in °C, *x*_2_ = UV in mW∙cm^−2^, *x*_3_ = pH. Multiple linear regression interpolation parameters were *R*^2^ = 0.97 and *Q*^2^ = 0.92. Here, *R*^2^ represents the fraction of the variation of the response explained by the model and *Q*^2^ is the fraction of the variation of the response that can be predicted by the model. More generally, *R*^2^ and *Q*^2^ provide the summary of the fit of the model: *R*^2^ is an overestimated and *Q*^2^ an underestimated measure of the goodness of the fitting of the model. Both of these values are rather close to 1, thus indicating that the regression model provides an excellent description of the relationship between the independent variables and the response. The fitting equations can be plotted in an isoresponse plot. In Fig. 4 the effect of each experimental parameter on a single variable can be clearly appreciated, as well as the great potential of the DoE technique for the deconvolution of the single contributions within a multisensing device.

To further support the statistical model, a cross-validation process was carried out. This process is a model validation technique useful to assess how the outcome of a chemometric model can be generalized to an independent data set. To this purpose, the software generated some new experiments to be done, and also predicted the result that should be obtained experimentally. The model can be considered valid if the obtained experimental results for these further runs are adherent to the predicted result with 95% confidence. In our case, the obtained experimental results were closely co-related with the predicted data, meaning that the chemometric model that we developed is robust and valid (see Table S2 in the Supplementary Information).

The chemometric model presented in this section allows to confidently predict the response of the proposed multisensor, based on a ZnO/gold junction, in terms of current, resistance and capacitance, also allowing the distinction between contributions of different input variables.

## Discussion

We demonstrated a powerful method to understand and then predict the behavior of a semiconductor/metal junction in a multisensing approach. This work is also a clear proof-of-concept about the fabrication of a real-time measurement device based on single ZnO μ-wire/gold junction, which allows multifunctional sensing. Furthermore, the ease of the multisensor assembly, based on EIBJ and DEP techniques, ensures the reproducibility and the re-use of the final device. These features, along with the capability of testing and predicting the device in a wide range of experimental conditions, are all key features for sensors development. Specifically, a quick prediction of the sensor output, in different experimental scenarios (i.e., the behavior of the ZnO/gold junction under different stimuli), can be obtained without the need of testing all of them. By changing the mathematical boundary conditions, plenty of distinct experimental situations can be modeled prior to or alternatively to experimental measurements.

It is noteworthy that modeling the behavior of this multisensing device is a complex multidisciplinary task. Nevertheless, our simplified model, with its assumptions and approximations, is capable to faithfully and accurately reproduce the experimental curves. This fact underlines the robust predictive potentialities of our multiparametric sensor model in different experimental conditions. Moreover, a multivariate chemometric investigation further confirmed the model and it was also useful for the formulation of the three equations that correlate the measured responses with the individual external stimuli.

These results will underpin future studies about additional ZnO sensing capabilities, e.g. molecules or mechanical pressure detection. These studies can be well identified and predicted, thus being completely incorporated in our multisensing approach. Broadening the potentialities of our multisensor can lead to a leading edge and resourceful device that, if embedded in a living cell culture or organ-on-a-chip, can help to reveal and understand the complex mechanisms occurring in the cellular microenvironment. Even further, the high versatility of ZnO material applied in energy storage^37^, photo-conversion^38^, and nanogenerator^4^,^5^,^6^ together with multi-parameters sensitivity can become a base for highly multifunctional and smart self-powered nanosensors.

## Methods

### ZnO μ-wires synthesis

The ZnO μ-wires were synthesized by slowly dropping 3.35 g of potassium hydroxide (60 mmol, Merck KGaA, Darmstadt, Germany, dissolved in 10 mL water) into 1.48 g zinc nitrate hexahydrate Zn(NO_3_)_2_ · 6H_2_O (5 mmol, Sigma-Aldrich S.r.l. Milan, Italy in 10 mL of bidistilled water) under vigorous stirring, as also reported in Cauda *et al*.^15^. The transparent solution was then transferred in a closed teflon vessel and it was placed in an oven at 70 °C for 5 h. Then, the formed ZnO μ-wires were collected by filtration, washed thoroughly with water until neutral pH was reached, and dried in air at 60 °C. Post-grafting with aminopropyl groups on the ZnO μ-wires was carried out with 10 mol% of the functional moiety with respect to ZnO molar amount. In detail, 250 mg (3.075 mmol) of ZnO μ-wires was outgassed for 2 h in a round flask connected to a Schlenk line. Then, the atmosphere was changed to nitrogen, 10 mL of dry toluene and 0.307 mmol of aminopropyltrimethoxysilane (APTMS; 55.04 mg) were added, and the solution was refluxed for 24 h under nitrogen. The functionalized μ-wires were washed with acetone and isopropanol and then dried at 60 °C overnight.

### Characterization

Field emission scanning electron microscopy (FESEM, ZEISS Dual-Beam Auriga) and X-ray diffraction with an X’Pert diffractogram (CuKα = 1.54 Å) in Bragg-Brentano configuration were used for material characterization (see Fig. 1d and Supplementary Figs S1 and S4). Fourier transmission infrared (FTIR) spectroscopy (see Supplementary Fig. S2) was carried out in attenuated total reflectance (ATR) on a Bruker Equinox 55 (spectra are baseline subtracted).

### Gold electrode chip fabrication

The chips used for assembling the single ZnO μ-wires were obtained by conventional photolithography. Four gold wires (25-nm thin, 5-mm long, and 300-μm wide) were fabricated on a Si wafer covered with 200 nm of silicon dioxide and a 5 nm titanium as adhesion layer for gold. From each gold wire two micrometric facing electrodes were repeatedly obtained by a fine controlled electromigration-induced break junction (EIBJ) using a custom hardware/software modular embedded system^32^,^39^. The whole electric platform consisted of a central silicon chip (5 × 5 mm) with the gold electrode-array, bonded to a customized printed circuit board (PCB, 16 × 25 mm). The bonding wires were incorporated in a 1k-epoxy casting resin ring, which was used for protecting and insulating the bonding wires and confining the ZnO wire suspension during the deposition.

One drop of amino-functionalized ZnO μ-wires in isopropanol (0.8 mg∙mL^−1^) was dispensed on the chip. DEP was carried out at 1 MHz AC signal and 3 V_pk-pk_ (sinusoidal waveform, offset 0 V) until the complete evaporation of the solvent. Electrical characterizations were performed through a precision source/meter unit for DC analysis (−1 V; +1 V) and an impedance analyzer for the AC measurements (5–100 kHz) both by Keysight Technologies. Both instruments were interfaced with a LabVIEW software for customized measurements acquisition.

### Multi-sensor measurement setup

The multisensing measurements under UV-VIS light, pH and temperature variations on the chip containing the ZnO/gold junctions were carried out using a custom-assembled setup in order to vary, individually or simultaneously, each stimulus (i.e. UV-VIS light, pH and T). An opaque cage sheltered the assembled sensing device from environmental light. A medium-pressure mercury lamp (LC8 Lightingcure^TM^ by Hamamatsu supplied with 8 mm light guide) tuned the UV-VIS illumination. The chip responsivity to different pH values was tested by spilling distinct drops (10 μl each) on the chip surface, then drying under nitrogen flux after few tenths of seconds. Hydrochloric acid (HCl) with molarities in the range 10 μM– 10 mM was used for acidic pH experiments (from pH 2 to 5), while sodium hydroxide (NaOH) with molarities between 10 μM–100 mM was chosen for the alkaline ones (from pH 9 to 13). The device was furthermore positioned on an heating plate and connected to a thermocouple with instantDAQ technology (National Instruments USB-TC01) to perform real-time temperature analysis.

The DC electrical characterization was performed through a precision source/meter unit (B2912A by Keysight Technologies) in the voltage range between −1 and +1 V. A precision impedance analyzer (4294a by Keysight Technologies) was instead used for the AC characterization in the frequency range 5 kHz–100 kHz. Both the instruments were interfaced with a computer using LabVIEW software for customized measurements acquisition.

The Design of Experiment (DoE) was carried out by means of a D-Optimal design, assisted with a MODDE software (version 7.0.0.1, Umetrics) and 18 experiments were considered.

## Additional Information

**How to cite this article**: Miccoli, B. *et al*. One-Dimensional ZnO/Gold Junction for Simultaneous and Versatile Multisensing Measurements. *Sci. Rep.* **6**, 29763; doi: 10.1038/srep29763 (2016).

## Supplementary Material

Supplementary Information

## Figures and Tables

**Table 1:** Multifunctional sensor specifications.

Studied Range	Sensitivity	Resolution			
*UV-VIS light*					
0–450 mW∙cm^−2^	0.03 μA∙mW^−1^∙cm^2^ between 0–50 mW∙cm^−2^	0.14 mW∙cm^−2^			
	0.01 μA∙mW^−1^∙cm^2^ between 50–200 mW∙cm^−2^				
	1.70∙10^−4^μA∙mW∙cm^−2^ above 200 mW∙cm^−2^				
*Temperature*					
0–66 °C	0.10 μA °C^−1^ between 22–40 °C	1 °C			
	0.33 μA °C^−1^ between 40–66 °C				
*pH*					
2–13	−4.50 nA∙pH^−1^ between pH 2–pH 5	1 unit			
	−4.17 nA∙pH^−1^ between pH 9–pH 13				

Sensitivity, resolution and analyzed UV-VIS light, temperature and pH ranges for the developed multiparametric sensor based on single ZnO μ-wire.
