# Supplementary material for: One-Dimensional ZnO/Gold Junction for Simultaneous and Versatile Multisensing Measurements
Source: Sci Rep. 2016 Jul 13;6:29763. doi: 10.1038/srep29763 (PMC4942824; doi:10.1038/srep29763)
Supplement: Supplementary Information [file srep29763-s1.pdf]

# Supplementary Information

## One-Dimensional ZnO/Gold Junction for Simultaneous and Versatile Multisensing Measurements.

*Beatrice Miccoli[1§], Valentina Cauda[2,3*§], Alberto Bonanno[2], Alessandro Sanginario[2], Katarzyna Bejtka[2], Federico Bella[3], Marco Fontana[3], Danilo Demarchi[1,2]*

[1] Department of Electronics and Telecommunication, Politecnico di Torino, Corso Duca degli Abruzzi 24, Torino 10129, Italy

[2] Center for Space Human Robotics@PoliTo, Istituto Italiano di Tecnologia, Corso Trento 21, Torino 10129, Italy

[3] Department of Applied Science and Technology, Politecnico di Torino, Corso Duca degli Abruzzi 24, Torino 10129, Italy

[§]These authors have equally contributed to the work

## Supplementary Figures

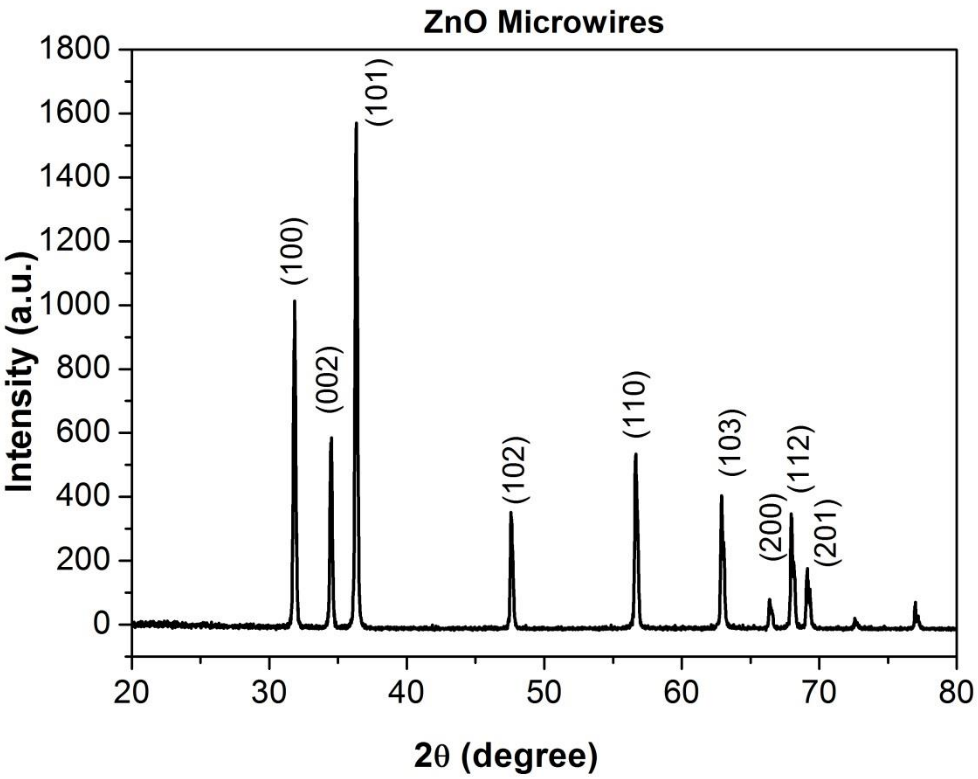


**Supplementary Figure 1| X-ray diffraction pattern of ZnO microwires.** The X-ray diffraction pattern shows the reflection typical of a wurtzite crystalline structure of the microwires (JCPDS 80–0074, a = 0.3253 nm, c = 0.5215 nm, hexagonal symmetry, space group P63mc). In addition, the sharp diffraction peaks indicate that the product has a high purity and high degree of crystallinity.

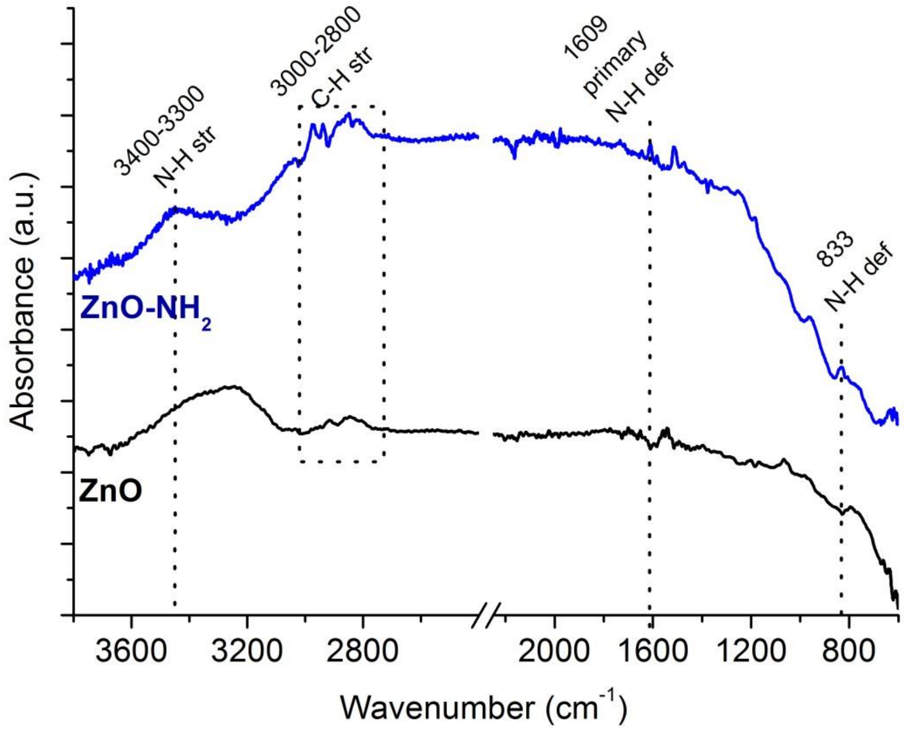


**Supplementary Figure 2| Fourier-Transform Infrared Spectroscopy.** The bare ZnO µ-wires (in black) show at 964 and 796 $cm^{-1}$ the asymmetric bending and stretching vibration of Zn-OH, respectively. A broad band from 3600 to 3100 $cm^{-1}$ corresponds to water and hydroxyl groups on the µ-wire surface. Similarly, the peak at 1629 $cm^{-1}$ indicates the bending modes of the water molecules adsorbed on the surface of the ZnO material. In the ZnO-$NH_2$ spectrum (in blue), the presence of amine groups in indicated by the deformations of primary amine (N-H) at both 833 and 1609 $cm^{-1}$. The band between 3500 and 3300 $cm^{-1}$ corresponds to the N-H stretching vibration, from 3000 to 2800 $cm^{-1}$ to the stretching vibration of the C-H groups, belonging to the propyl chain.

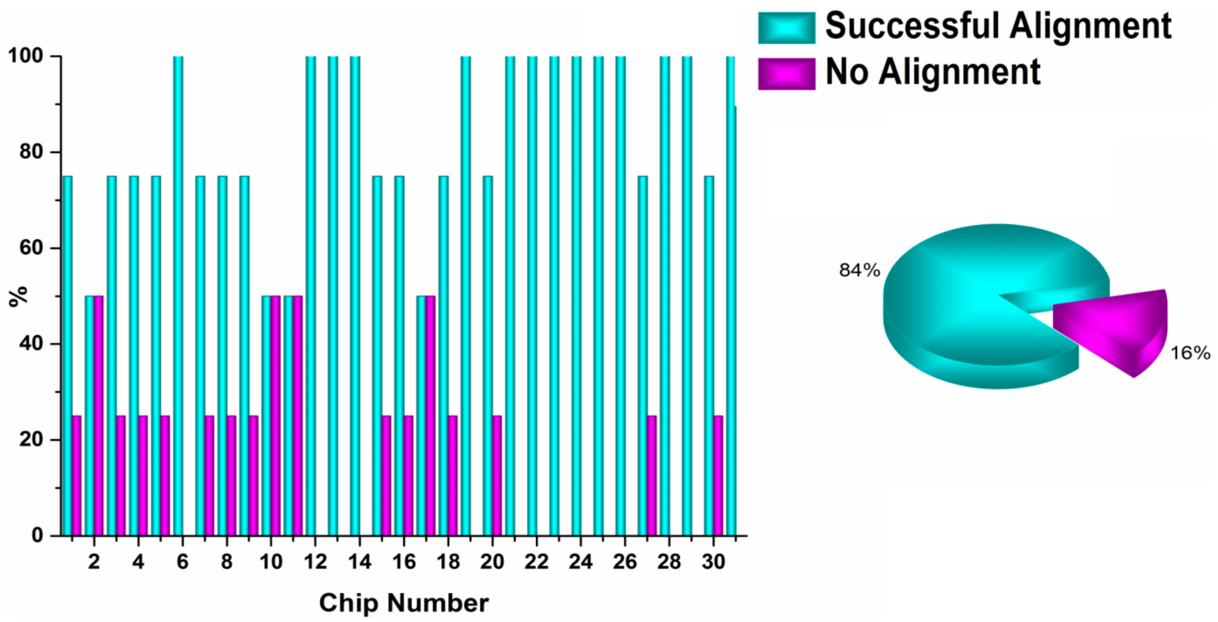


**Supplementary Figure 3| Successful ZnO μ-wire alignment percentage.** The results of the 31 dielectrophoresis experiments performed on the custom-fabricated chips. A total of 124 electrodes pairs, four for each chip, are tested. The confirmation about the correct ZnO μ-wire alignment is performed at first optically, through an optical microscope, and then electrically i.e. no open circuit condition must be detected. For each chip, the successful alignment percentage is computed considering the number of the chip's electrodes pairs presenting a correct ZnO μ-wire alignment. The alignment is considered unsuccessful if no ZnO μ-wire is bridging the two Au electrodes, while 100% successful alignment is achieved if all the four gaps of the chip are correctly bridged. The 84% of the electrodes pairs tested exhibit correct ZnO μ-wire deposition.

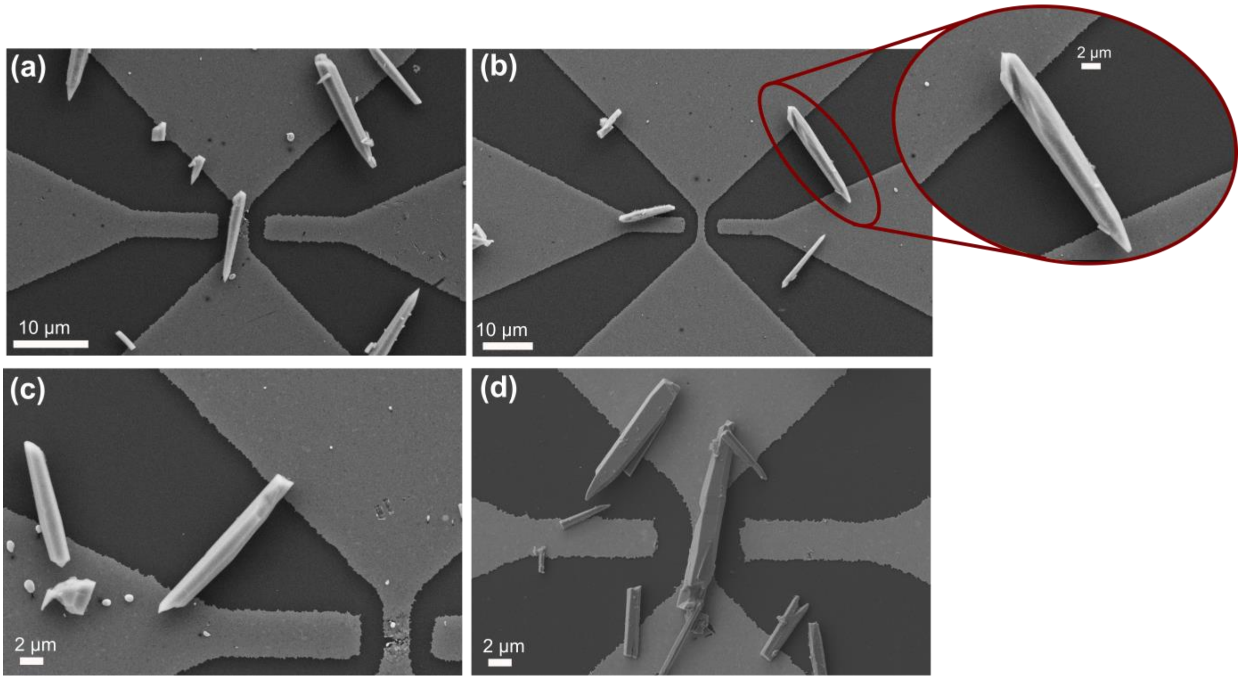


**Supplementary Figure 4 | Electron Microscope Characterization.** The results of four different DEP experiments are investigated by FESEM. In each of the four pictures, a single ZnO µ-wire is bridging either the two gold electrodes created by EIBJ (a and d) or those fabricated by optical lithography (b and c).

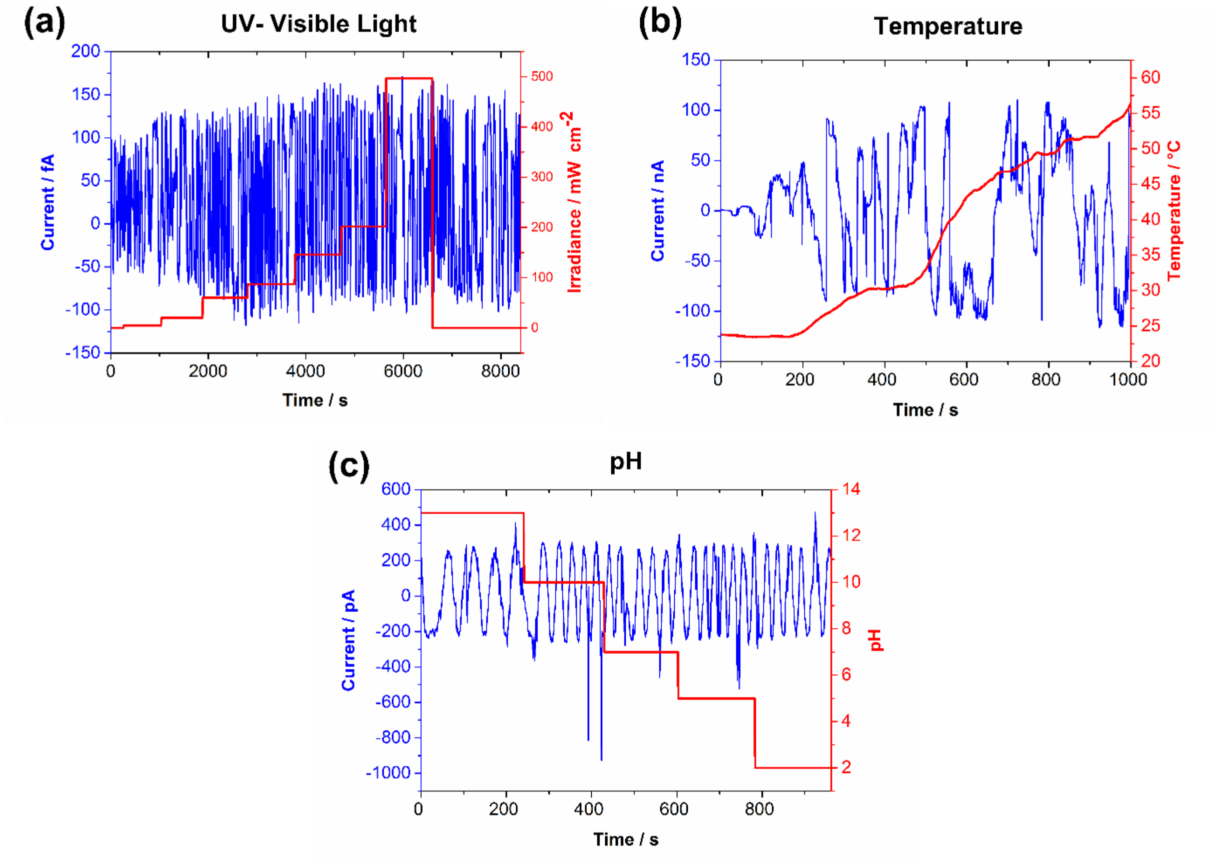


**Supplementary Figure 5| Control Measurements.** One couple of gold electrodes, with no ZnO μ-wire bridging them, is electrically characterized upon external variations of UV-Visible light, temperature and pH (red curves). The applied voltage is 1 V. As expected, just noise is measured (blue curves), since an open circuit condition is present, and no fluctuations correlated to the variation of the external stimuli are registered.

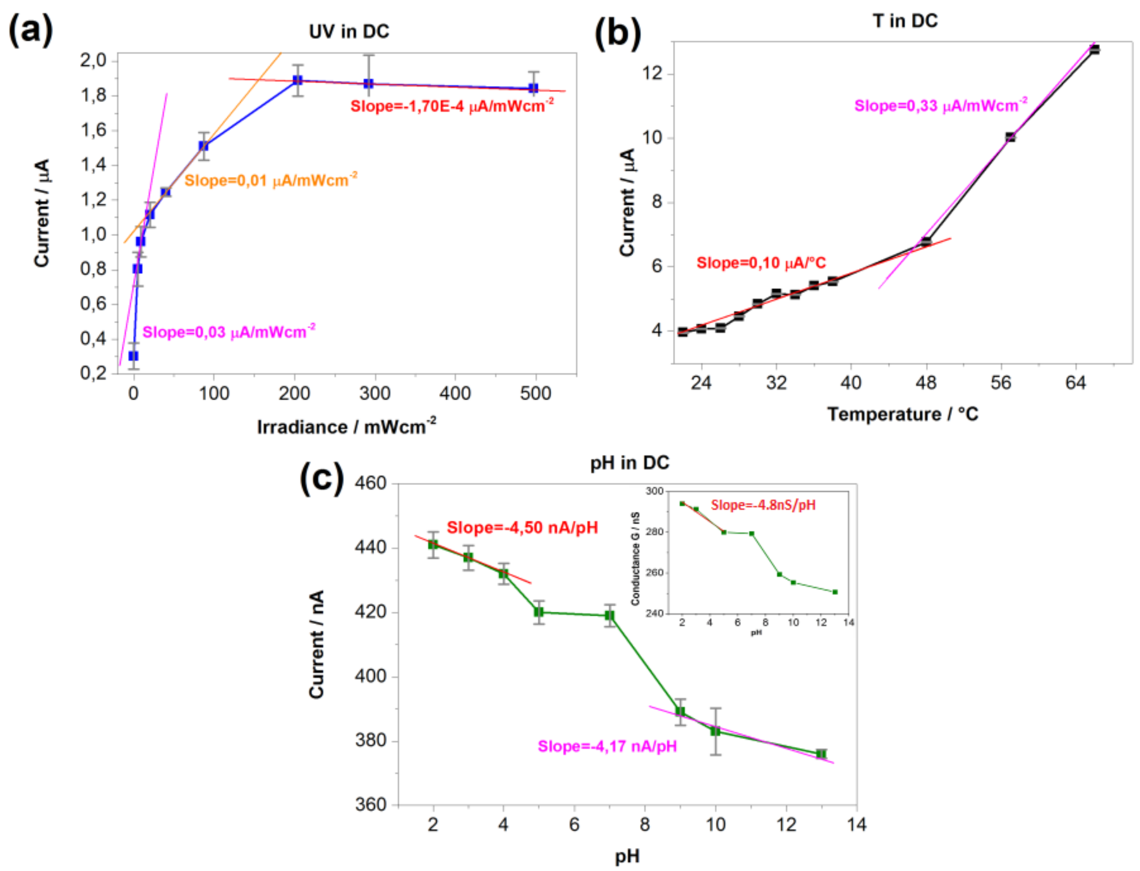


**Supplementary Figure 6| DC electrical behavior of the ZnO/gold junctions under different external stimuli.** The DC electrical behavior of the ZnO/gold junctions measured under (a) UV-visible light irradiation, (b) temperature, and (c) pH variations. The three graphs are obtained in DC domain, measuring the current at 1 V. The sensor sensitivities, for each of the three stimuli considered independently, are extracted from the slope of the line tangent to the experimental curves in specific ranges. For UV-visible light, temperature and pH external variations, the sensitivity exhibits a non linear characteristic identifying preferred experimental ranges of higher sensitivity. For UV-Visible light external variations (a), the sensitivity shows higher values for lower irradiance (0.03 $\mu A \cdot mW^{-1} \cdot cm^2$ in the range 0-50 $mW \cdot cm^{-2}$ and 0.01 $\mu A \cdot mW^{-1} \cdot cm^2$ in the range 50-200 $mW \cdot cm^{-2}$), while it strongly decreases (up to $1.70 \cdot 10^{-4}$ $\mu A \cdot mW^{-1} \cdot cm^2$) above ≈200 $mW \cdot cm^{-2}$. Temperature variations (b), instead, point out a sensitivity increase (from 0.10 µA·°C to 0.33 µA·°C) for temperatures above ≈48

°C. Interestingly, pH changes (c) show almost the same sensitivity, 4.50 nA·$pH^{-1}$ and 4.17 nA·$pH^{-1}$ in absolute value, for strong acid and basic pH, respectively.

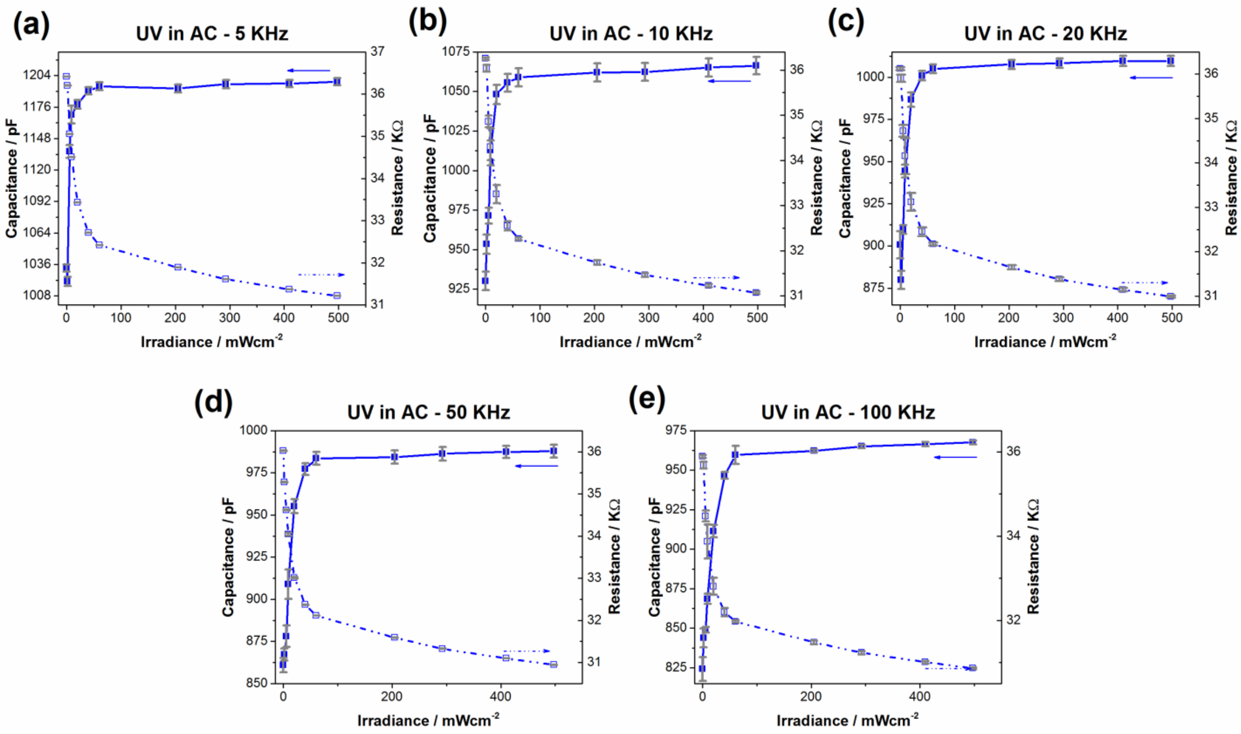


**Supplementary Figure 7| AC electrical characterization varying UV-visible irradiation.** The AC electrical behavior of ZnO/gold junctions measured under UV-visible light irradiation variation (capacitance is on the left y-axis and resistance on the right y-axis) obtained at frequencies of (a) 5 kHz, (b) 10 kHz, (c) 20 kHz, (d) 50 kHz and (e) 100 kHz.

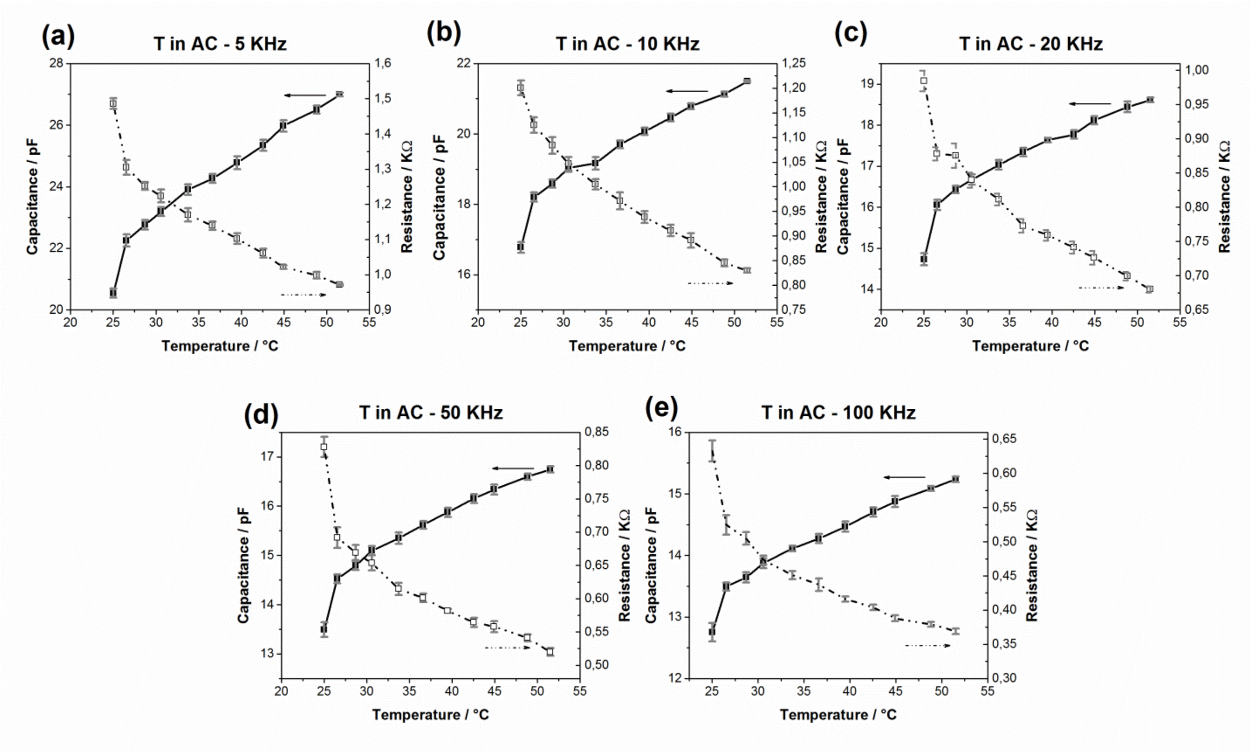


**Supplementary Figure 8| AC electrical characterization varying temperature.** The AC electrical behavior of the ZnO/gold junction measured under temperature variation. The graphs are AC measurements with capacitance (y-axis on the left) and resistance (y-axis on the right) values obtained at different frequencies of (a) 5 kHz, (b) 10 kHz, (c) 20 kHz, (d) 50 kHz and (e) 100 kHz.

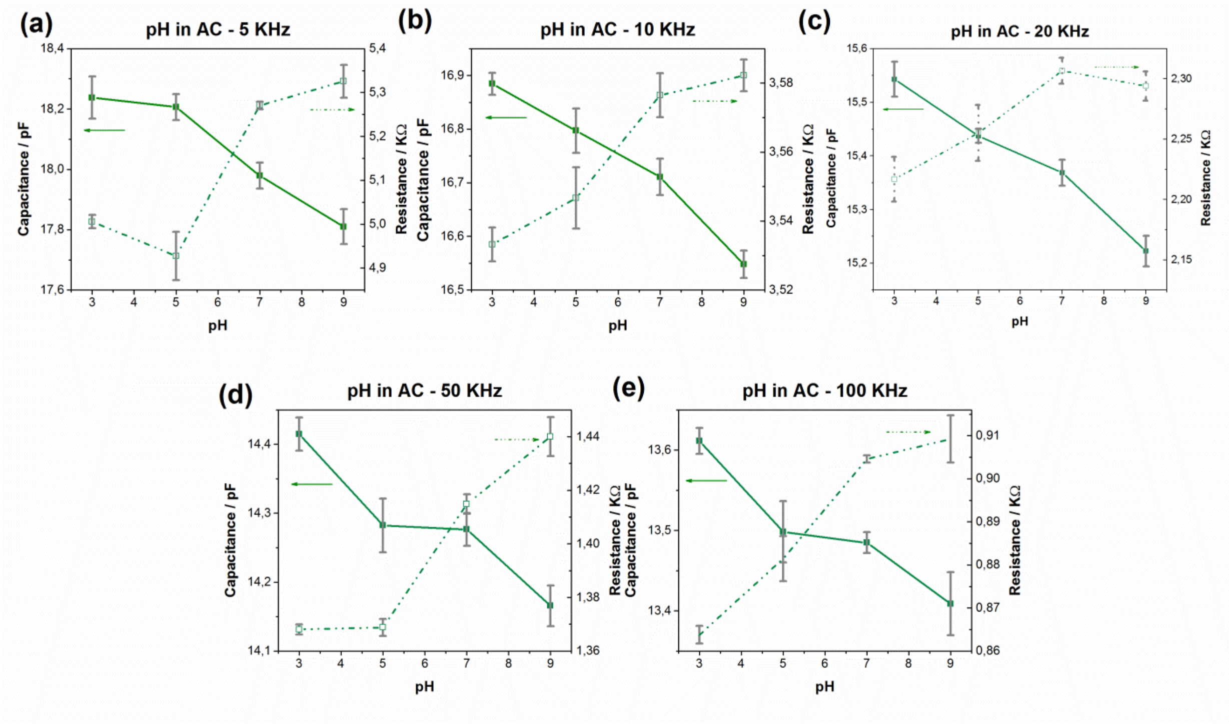


**Supplementary Figure 9| AC electrical characterization varying pH.** The electrical behavior of the ZnO/gold junction measured under pH variation (capacitance is on the left y-axis and resistance on the right y-axis) at different frequencies (a) 5 kHz, (b) 10 kHz, (c) 20 kHz, (d) 50 kHz and (e) 100 kHz.

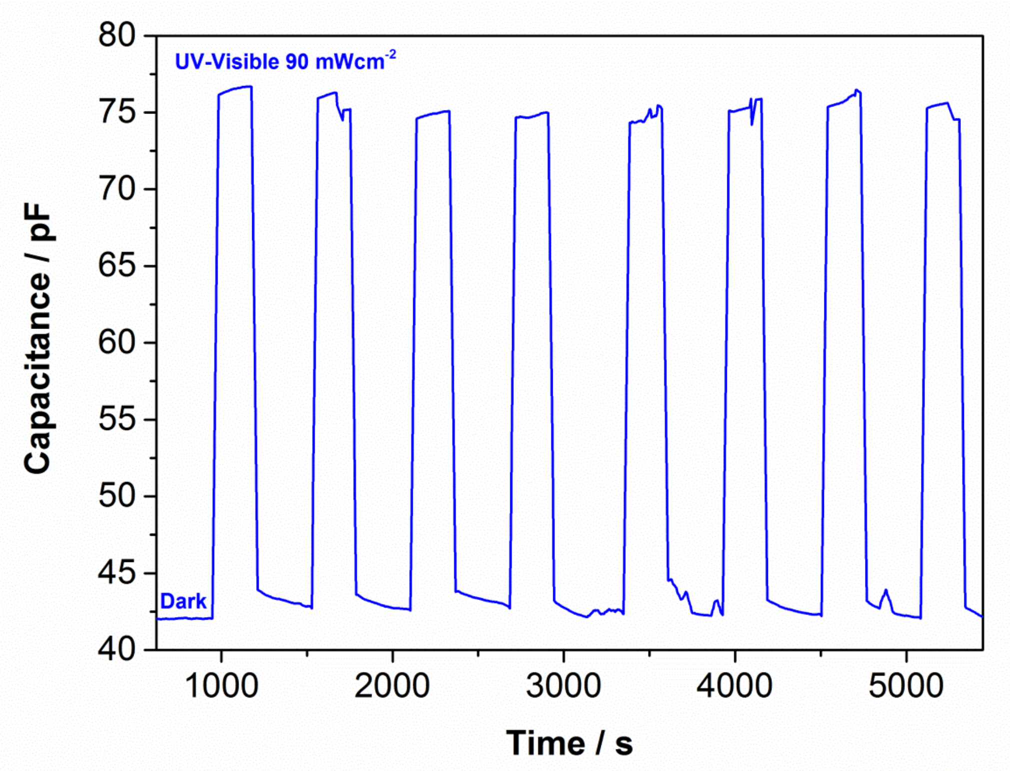


**Supplementary Figure 10 | UV-Visible on/off cycles.** The UV-visible on/off cycles performed on a single ZnO µ-wire/gold junction are shown. The results represent the capacitance variation, at 100 KHz, when the UV-visible light, at 90 mW·cm$^{-2}$, is switched on and off for eight times.

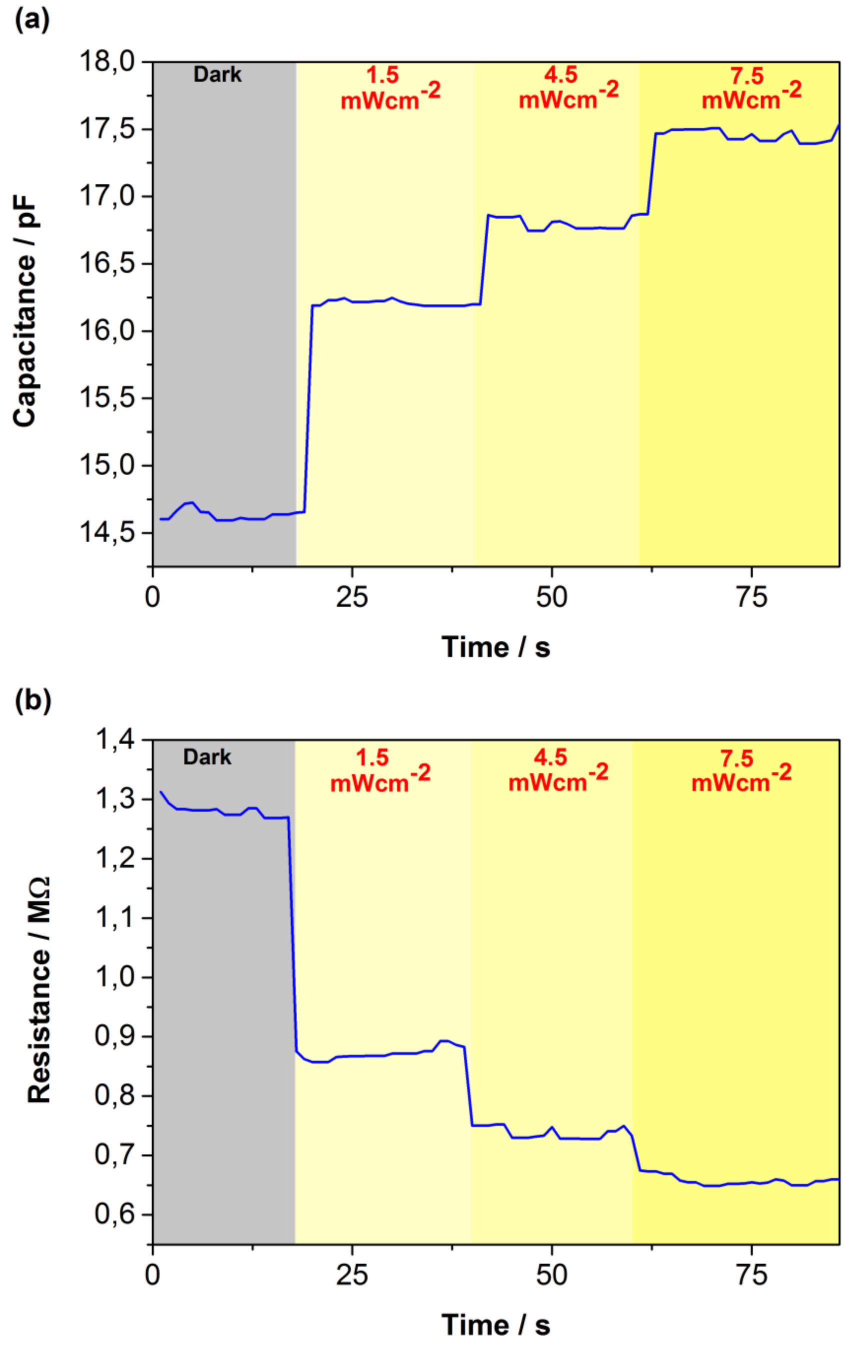


**Supplementary Figure 11| Time dependent AC behavior varying UV-visible light.** The time-dependent AC behavior of the ZnO/gold junction at increasing UV-visible light irradiation: (a) capacitance and (b) resistance obtained at 50 kHz.

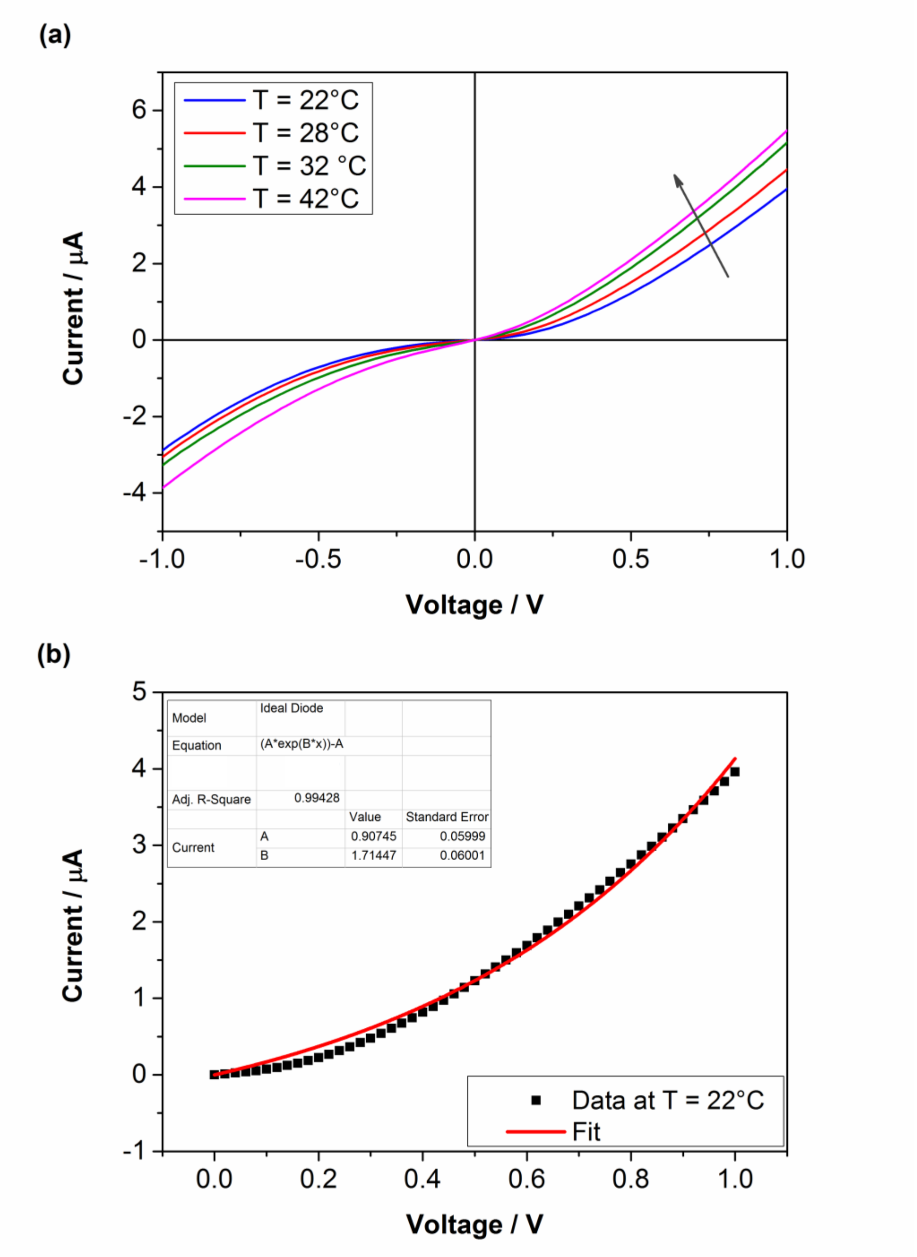


**Supplementary Figure 12| ZnO/gold junction IV curves and fit with the ideal diode law.** (a) The I-V characteristics the ZnO/gold junction measured at varying temperatures between −1 V and 1 V. Fit of the experimental curve at T = 22 °C with the ideal diode equation (b). The value of the saturation current and of the ideality factor of the Zn-gold Schottky junction are obtained from fitting parameters A ($I_S$=A) and B (n=q/B$K_B$T), respectively.

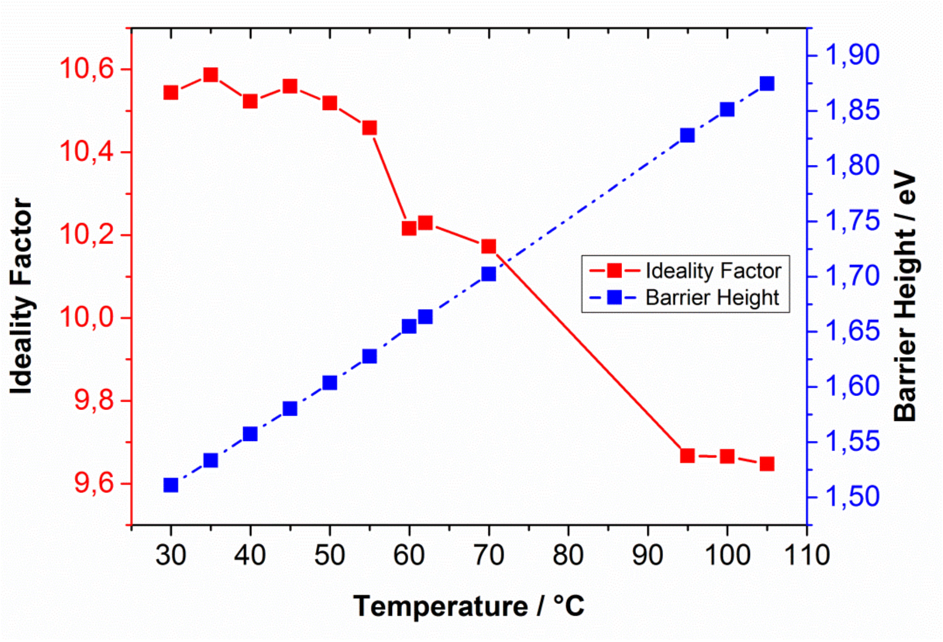


**Supplementary Figure 13| ZnO/gold junction ideality factor and barrier height.** Behavior of the ZnO/gold junction ideality factor (y-axis on the left) and the barrier height (y-axis on the right) under temperature variation. Both the data are extrapolated from the fit of the experimental I-V curves at different temperatures with the ideal diode equation.

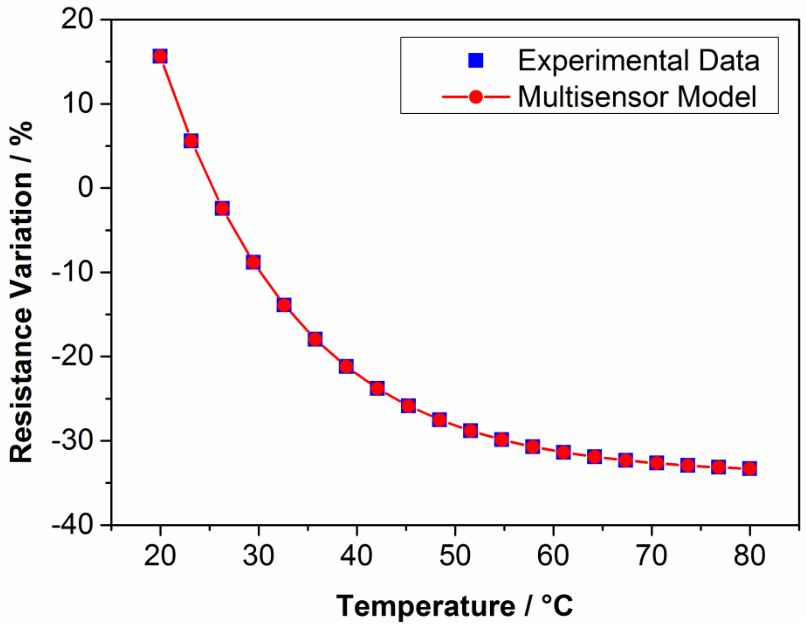


**Supplementary Figure 14| Fit of the resistance variation upon temperature with the multiparametric sensor model**. Resistance percentage variation as function of temperature at 5 kHz. The behavior predicted by the multiparametric sensor model (red curve) is compared

with the experimental data (blue squares) The boundary conditions are pH = 7 and UV-visible irradiance = 0 mW $cm^{-2}$.

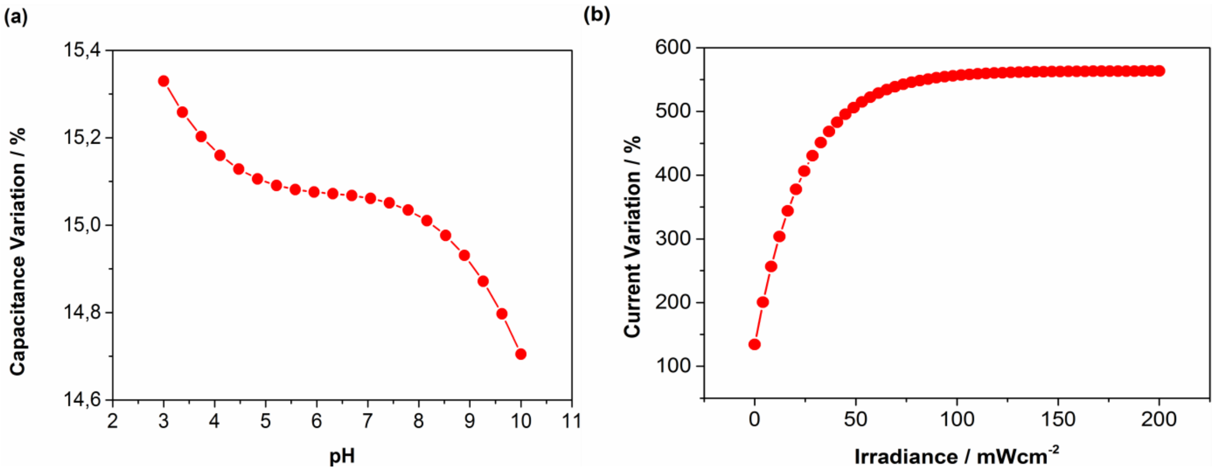


**Supplementary Figure 15| Fit of the capacitance and current variations upon, respectively, pH and UV-visible irradiation with the multiparametric sensor model**. (a) Capacitance percentage variation as a function of pH predicted by the multisensory model (red curve) at 5 kHz. The value of UV-visible irradiance is fixed at 1.49 $mWcm^{-2}$ while the temperature at 24 °C. (b) Current percentage variation as a function of UV-visible irradiance predicted by the multisensor model (red curve). The value of pH is fixed at 4 while the temperature at 55 °C.

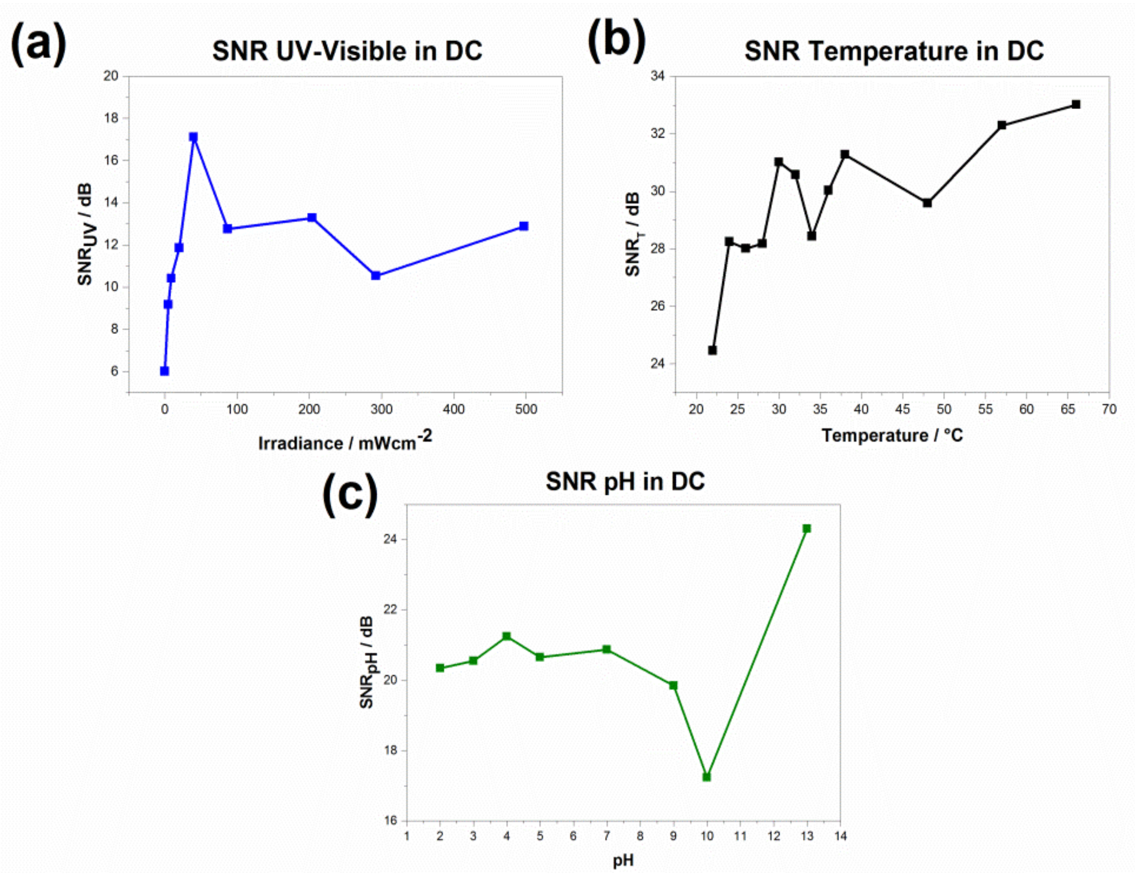


**Supplementary Figure 16| SNR in DC.** SNR computation for UV-Visible light (a), temperature (b) and pH (c) measurements in DC when the external stimuli are independently applied.

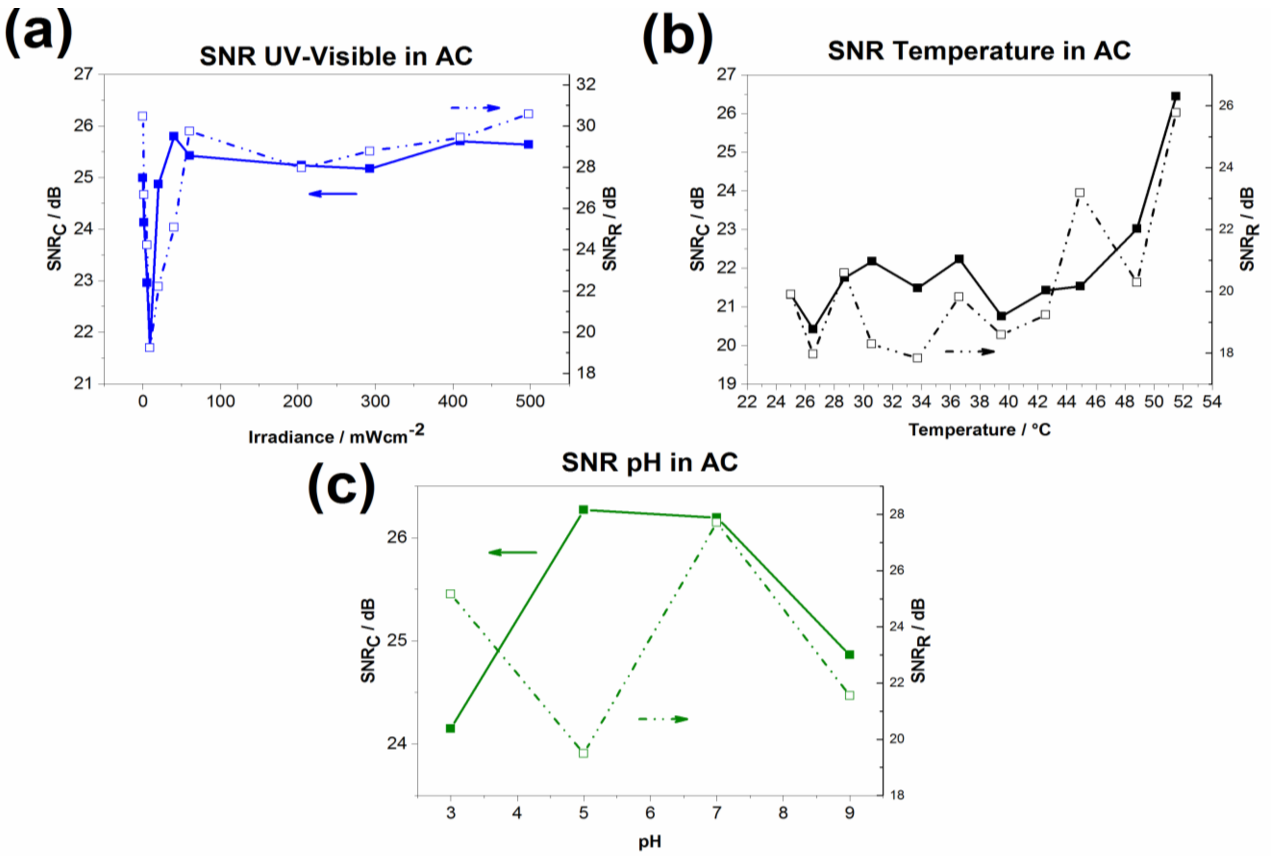


**Supplementary Figure 17| SNR in AC.** SNR computation for UV-Visible light (a), temperature (b) and pH (c) measurements in AC when the external stimuli are independently applied. The SNR is computed for both resistance (dotted lines) and capacitance (solid lines) measurements.

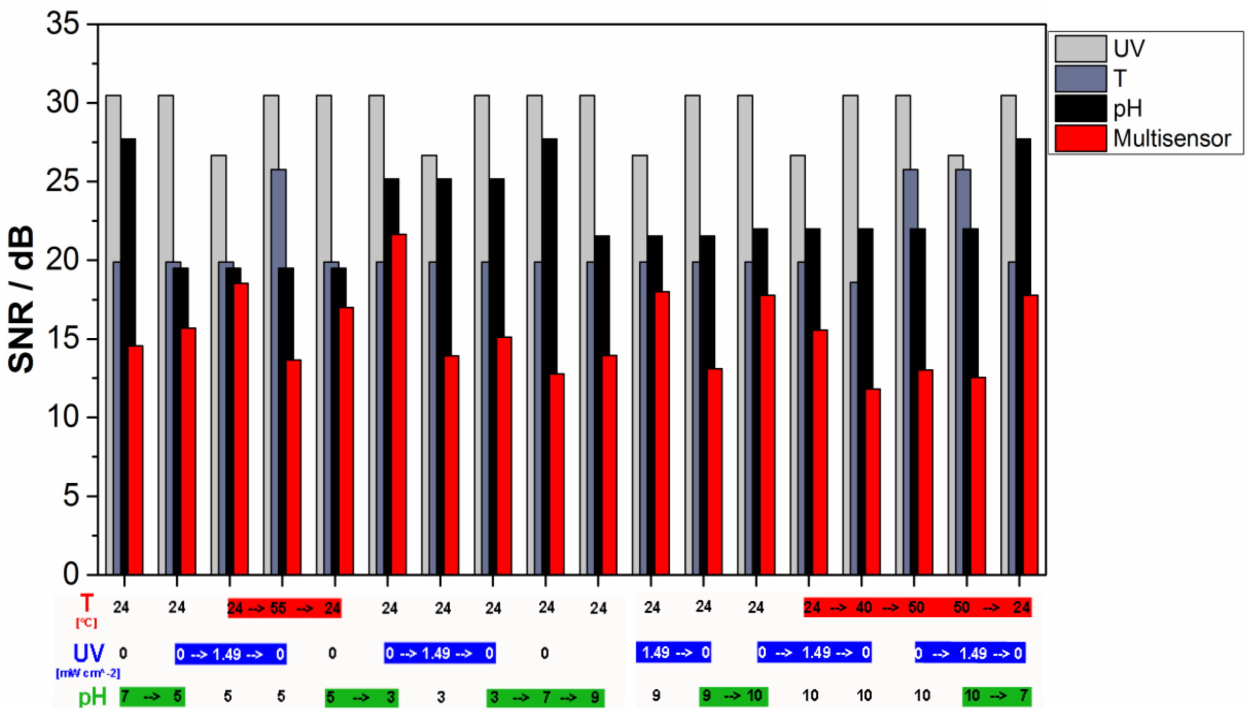


**Supplementary Figure 18| SNR Comparison Between Multiple and Individual Sensing.** Comparison of the SNR measured during resistive multisensory experiments (red bars) and the one measured during the individual sensing of UV-Visible light (light grey bars), temperature (dark grey bars) and pH (black bars) variations, at the same experimental points. The legend below the column plot lists all the different tested cases.

## Supplementary Tables

**Supplementary Table 1: Multiparametric sensor modeling equations.** Equations of the functions reported in Figure 2 (red lines) fitting the experimental data collected for the current % variation (*f*, *g*, *h*), the capacitance % variation (*f'*, *g'*, *h'*), and the resistance % variation (*f''*, *g''*, *h''*).

| Modeling Function | Adj. R-Square |
|---|---|
| *Current modeling functions* | |
| $f(x_{UV})= -429.220 \cdot \exp(-x_{UV} \cdot 0.041)+432.824$ | 0.963 |
| $g(x_T)= 18.370 \cdot \exp(x_T \cdot 0.0407)-44.419$ | 0.989 |
| $h(x_{pH})= 3.714+1.512 \cdot x_{pH} -0.501 \cdot x_{pH}^2+0.023 \cdot x_{pH}^3$ | 0.945 |
| *Capacitance modeling functions* | |
| $f'(x_{UV})= -111.367 \cdot \exp(- x_{UV} \cdot 0.134)+121.883$ | 0.999 |
| $g'(x_T) = -267.410 \cdot \exp(- x_T \cdot 0.018)+170.135$ | 0.988 |
| $h'(x_{pH})= 5.866-2.723 \cdot x_{pH} +0.428 \cdot x_{pH}^2-0.022 \cdot x_{pH}^3$ | 0.998 |
| *Resistance modeling functions* | |
| $f''(x_{UV})= -45.000-20.414*\ln(x_{UV} + 0.400)$ | 0.997 |
| $g''(x_T)= 207.748 \cdot \exp(- x_T \cdot 0.07159)-34.006$ | 0.996 |
| $h''(x_{pH})= -5.052-0.744 \cdot x_{pH} +0.380 \cdot x_{pH}^2-0.025 \cdot x_{pH}^3$ | 0.969 |

## Supplementary Table 2: Cross-validation data for the multivariate chemometric approach.

| *T* | *UV* | *pH* | *Capacitance (predicted)* | *Capacitance (measured)* |
|---|---|---|---|---|
| 24 | 0 | 5 | -0.0723 +/- 0.01 | -0.066 |
| 24 | 0.14 | 7 | 15.954 +/- 1.44 | 16.913 |
| 33.75 | 0 | 7 | 21.492 +/- 1.74 | 21.299 |
| 24 | 1.49 | 3 | 16.112 +/- 1.64 | 15.003 |
| *T* | *UV* | *pH* | *Resistance (predicted)* | *Resistance (measured)* |
| 24 | 0 | 5 | 4.702 +/- 0.52 | 5.118 |
| 24 | 0.14 | 7 | -22.685 +/- 2.40 | -24.156 |
| 33.75 | 0 | 7 | -14.034 +/- 1.22 | -14.993 |
| 24 | 1.49 | 3 | -46.802 +/- 2.77 | -46.326 |
| *T* | *UV* | *pH* | *Current (predicted)* | *Current (measured)* |
| 24 | 29 | 7 | 321.23 +/- 5.22 | 325.806 |
| 24 | 58.54 | 7 | 359.44 +/- 5.73 | 354.839 |
| 24 | 146.35 | 7 | 430.87 +/- 6.18 | 432.258 |
| 32 | 0 | 7 | 31.19 +/- 1.62 | 30.409 |
| 24 | 0 | 5 | 0.26 +/- 0.07 | 0.239 |

**Supplementary Notes**

**Supplementary note 1. ZnO/gold junction ideality factor and barrier height.**

The ZnO/gold junction ideality factor and barrier height are extracted from the experimental IV curves in order to qualitatively analyze the goodness of the Schottky behavior. At first, the experimental I-V curves are fitted with the ideal Schottky diode equation (Supplementary Equation 1)[2] in the voltage range 0 V – 1 V.

$$I = I_S\left[e^{\frac{qV}{nk_BT}} - 1\right] \qquad (1)$$

where $q$ is the electron charge, $V$ is the bias voltage, $n$ is the ideality factor, $k_B$ is the Boltzmann constant and $T$ is the temperature expressed in K.

The value of the saturation current and of the ideality factor can then be extracted from fitting parameters as shown in Supplementary Fig. 6b for the I-V measurements performed at 22 °C. To estimate the value of the barrier height, $\Phi_B$, the Arrhenius plot of the saturation current equation (Supplementary Equation 2) is exploited[1].

$$I_S = A^{**}ST^2e^{\left(\frac{-q\phi_B}{k_BT}\right)} \quad \rightarrow \quad ln\left(\frac{I_S}{T^2}\right) = ln(A^{**}S) - \frac{q\phi_B}{K_BT} \qquad (S2)$$

where $A^{**}$ is the effective Richardson constant and $S$ is the diode area. The behavior of the ideality factor and of the barrier height extracted from the I-V curves for different temperatures are reported in Supplementary Fig. 7. The obtained ideality factor is higher than 1, which is the reference value for an ideal diode according to the thermionic emission model. This indicates that an important deviation from the theoretical model occurs in the experimental I-V curves. Nevertheless, the curve trends are in accordance with what reported in literature: as the temperature increases, the effective barrier height increases, since more electrons acquire enough energy to overcome the potential barrier, along with a decrease of the ideality factor[2].

**Supplementary note 2. Signal-to-Noise ratio estimation and comparison.**

The signal-to-noise ratio (SNR) is estimated according to the known formula: SNR = μ / σ, where μ is the signal mean, i.e. describing what is being measured, and σ is the noise standard deviation, i.e. representing noise and other interferences. They are calculated as follows:

$$\mu = \frac{1}{N}\sum_{i=0}^{N} x_i \tag{1}$$

$$\sigma = \sqrt{\frac{1}{N-1}\sum_{i=0}^{N-1}(x_i - \mu)^2} \tag{2}$$

In Supplementary Figs. S16-S17 the values of SNR, expressed in decibel, are reported for all the individual sensing both in DC (Supplementary Fig. S16) and in AC (Supplementary Fig. S17). As outlined in Supplementary Figs. S16 and S17, the SNR value is almost in the same range for all the measurements both in DC and in AC. Just the measurements when UV-Visible light is varying presents a slight deviation from the others but of little relevance. These can be considered quite appreciable results if it is considered that the measurements under different external stimuli are performed on three different chips, i.e., three different ZnO μ-wires, and in three different times.

To study the SNR behavior when multiple external stimuli are varied at the same time, the resistance curve is taken as an example. In Supplementary Fig. S18 it is outlined a column plot representing the SNR of the resistance during the multisensory measurements (red bars) and the one obtained, instead, during the individual sensing measures (greyscale bars), at under the same experimental conditions. As evident from Supplementary Fig. S18, the SNR of the multisensor measurements, despite being of the same order of magnitude, slight decreases from the value obtained during individual sensing. This may in principle be attributed to a cumulative effect of the noise associated to the variation of the three different external stimuli. Therefore, according to the known rules for error propagation, the

denominator in the SNR formula, i.e. the standard deviation representing the noise, will increase leading to a reduction of the global SNR of the multisensor.

**Supplementary note 3. Multiparametric sensor modeling.**

The slight deviation of the multisensing equations $I_{DC}$, $C_{AC}$, $R_{AC}$ from the experimental data in Fig. 3 in the main text appears when more than one stimulus is applied simultaneously to the sensing junction. This deviation is due to the modeling linear combination, in which the fitting errors of each fitting function are linearly summed.

To prove the reconstruction capability of the presented multisensing module, an additional investigation of the proposed mathematical model was carried out.

At first, two stimuli among UV-visible irradiation, temperature and pH are fixed, e.g. UV-visible and pH, and will be then addressed as boundary conditions. The remaining one, e.g. the temperature, is varied in the range 20 – 80 °C and its behavior is reconstructed according to the multisensor model in order to predict the sensor output at different temperatures. Thus the temperature is considered as independent variable, $x_T$, in the multiparametric sensor equations (equation 3 in the main text) and - as a representative example - we calculated the resistance output ($R_{AC}$) in the multisensory model as reported in Supplementary Equation 3 (the procedure is the same for all DC and AC outputs).

$$\begin{cases} x_{pH} = 7 \\ x_{UV} = 0 \\ R_{AC} = f''(x_{UV}) + g''(x_T) + h''(x_{pH}) \qquad x_T = [20, \ldots, 80] \end{cases} \tag{3}$$

where $x_{pH}$ corresponds to the fixed pH value, $x_{UV}$ is the fixed UV irradiance expressed in $mW \cdot cm^{-2}$, $x_T$ is the variable temperature expressed in °C. Equations f'', g'', h'' are the fitting functions reported in Supplementary Table 1. The output predicted by the multiparametric sensor model is thus shown in Supplementary Fig. 8 by the red dot curve. The obtained behavior is in line with the experimental one (blue square curve) since the applied boundary

conditions bring to zero the values of *f''(0)* and *h''(7)*, thus making the $R_{AC}$ function equal to the temperature fitting function, *g''*($x_T$), as also reported in Fig. 2e when only the temperature varies.

This first preliminary step is fundamental for the validation of the constructed mathematical model with respect experimental data. The same procedure can be repeated for any other multiparametric sensor equations $I_{DC}$, $C_{AC}$ or $R_{AC}$ or boundary conditions. For instance, the capacitance response of the sensor to a variable pH stimulus at fixed temperature ($x_T$ = 24 °C) and UV-irradiation ($x_{UV}$ = 1.49 mWcm$^{-2}$) is reported in Supplementary Fig. 9a.

Another prediction, in DC domain, is depicted in Supplementary Figure 9b considering a variable UV irradiation and fixed pH ($x_{pH}$ = 10) and temperature ($x_T$ = 55 °C).

In Supplementary Fig. 9 it can be appreciated that the trend of both curves are in complete accordance with the expected ones (see Fig. 2f and 2c in the main text). This fact underlines the robust predictive potentialities of the multiparametric sensor model in different experimental conditions.

## Supplementary References


1. R. Sharma, *Journal of Electron Devices* **2010**,8,286.
2. S. N. Das, J. P. Kar, K. Moon, in *Nanowires - Fundam. Res.* (Ed.: A. Hashim), InTech, **2011**, Ch.8.
